# Arterial spin labeling-based Z-maps have high specificity and positive predictive value for neurodegenerative dementia compared to FDG-PET

**DOI:** 10.1007/s00330-017-4784-1

**Published:** 2017-04-03

**Authors:** David Fällmar, Sven Haller, Johan Lilja, Torsten Danfors, Lena Kilander, Nelleke Tolboom, Karl Egger, Elias Kellner, Philip M. Croon, Sander C. J. Verfaillie, Bart N. M. van Berckel, Rik Ossenkoppele, Frederik Barkhof, Elna-Marie Larsson

**Affiliations:** 1 Department of Surgical Sciences, Radiology, Uppsala University, Uppsala, Sweden; 2 Department of Neuroradiology, University Medical Center Freiburg, Freiburg, Germany; 3 Faculty of Medicine, University of Geneva, Geneva, Switzerland; 4 Affidea CDRC - Centre Diagnostique Radiologique de Carouge, Carouge, Switzerland; 5 Department of Surgical Sciences, Nuclear Medicine and PET, Uppsala University, Uppsala, Sweden; 6 Hermes Medical Solutions, Stockholm, Sweden; 7 Department of Public Health and Caring Sciences, Geriatrics, Uppsala University, Uppsala, Sweden; 8 Department of Radiology and Nuclear Medicine, VU University Medical Center, Neuroscience Campus, Amsterdam, The Netherlands; 9 Department of Radiology, Medical Physics, Faculty of Medicine, Medical Center University of Freiburg, Freiburg, Germany; 10 Department of Neurology, Alzheimer Center Amsterdam, VU University Medical Center, Amsterdam, The Netherlands; 11 Institutes of Neurology and Healthcare Engineering, UCL, London, UK

**Keywords:** Neurodegenerative, Dementia, 18F-FDG, Arterial spin labeling MRI, Visual assessment

## Abstract

**Objective:**

Cerebral perfusion analysis based on arterial spin labeling (ASL) MRI has been proposed as an alternative to FDG-PET in patients with neurodegenerative disease. Z-maps show normal distribution values relating an image to a database of controls. They are routinely used for FDG-PET to demonstrate disease-specific patterns of hypometabolism at the individual level. This study aimed to compare the performance of Z-maps based on ASL to FDG-PET.

**Methods:**

Data were combined from two separate sites, each cohort consisting of patients with Alzheimer’s disease (*n* = 18 + 7), frontotemporal dementia (*n* = 12 + 8) and controls (*n* = 9 + 29). Subjects underwent pseudocontinuous ASL and FDG-PET. Z-maps were created for each subject and modality. Four experienced physicians visually assessed the 166 Z-maps in random order, blinded to modality and diagnosis.

**Results:**

Discrimination of patients versus controls using ASL-based Z-maps yielded high specificity (84%) and positive predictive value (80%), but significantly lower sensitivity compared to FDG-PET-based Z-maps (53% vs. 96%, *p* < 0.001). Among true-positive cases, correct diagnoses were made in 76% (ASL) and 84% (FDG-PET) (*p* = 0.168).

**Conclusion:**

ASL-based Z-maps can be used for visual assessment of neurodegenerative dementia with high specificity and positive predictive value, but with inferior sensitivity compared to FDG-PET.

***Key points*:**

• *ASL-based Z-maps yielded high specificity and positive predictive value in neurodegenerative dementia.*

• *ASL-based Z-maps had significantly lower sensitivity compared to FDG-PET-based Z-maps.*

• *FDG-PET might be reserved for ASL-negative cases where clinical suspicion persists.*

• *Findings were similar at two study sites.*

## Introduction

The prevalence of dementia is rapidly increasing, and the current prognosis is that 131.5 million individuals will be afflicted by 2050 [1], creating a great need for effective clinical diagnostic methods with high availability. Disease-modifying pharmaceutical agents may become available in the near future, which will increase the incentive for early diagnosis.

The classical role of neuroimaging in patients with suspected dementia has been to rule out treatable causes such as subdural haematomas and brain tumours. Modern neuroimaging also provides positive findings to support the clinically suspected diagnosis of neurodegenerative diseases such as Alzheimer disease’s (AD) or frontotemporal dementia (FTD). Patterns of brain atrophy on structural MRI become abnormal relatively late in the neurodegenerative disease process. Functional changes precede structural changes [2], and imaging of regional physiological parameters such as perfusion or glucose metabolism increases sensitivity and enables earlier diagnosis [3]. Glucose metabolism of the brain can be examined with [^18^F]-2-fluoro-2-deoxy-D-glucose (FDG) PET, which has proved to be a robust method for detecting patterns of regional deficits [4, 5].

Recently, cerebral perfusion analysis based on the MRI sequence arterial spin labeling (ASL) has been suggested as a biomarker for assessing the physiological state of the brain in patients with suspected neurodegenerative disease [6–10]. ASL can provide quantifiable perfusion maps in a few minutes, without injecting any radioactive material or contrast agent [11]. There is a strong association between perfusion and glucose metabolism in the brain, and patterns of ASL hypoperfusion have been shown to be highly similar to the patterns of hypometabolism in patients with neurodegenerative dementia [12–14]. If ASL can provide information similar to that obtained with FDG-PET, a fairly short MRI protocol that includes an ASL sequence could provide both morphological and physiological diagnostic information non-invasively.

Visual assessment of FDG-PET images requires a high level of experience and can be challenging, especially for novice readers. The images are often thresholded to show cortical areas with statistically significantly decreased metabolism in the individual patient compared to a database of healthy controls. This highlights areas with metabolism levels at the far left end of a standardized normal distribution curve. Such Z-score maps have been shown to improve the accuracy of diagnosing neurodegenerative disease [3, 15], especially for novice readers [16]. Currently, there are several commercially available software packages for FDG-PET analysis that allow Z-maps to be used in a clinical setting. These types of software commonly apply three-dimensional stereotactic surface projection maps (3D-SSP maps), originally introduced by Minoshima et al. [17].

While Z-maps are established for FDG-PET, they have only recently begun to be explored for ASL [18]. Due to the established perfusion-metabolism coupling in the brain, ASL-based Z-score maps should be similar to FDG-PET-based Z-score maps. MRI is routinely performed for imaging of neurodegeneration; and adding ASL would provide functional information in addition to the standard structural information. Also, ASL is of particular interest in the light of emerging specific amyloid and tau tracers for PET. Instead of performing two PET scans in a single patient, ASL might become a clinical biomarker of unspecific perfusion/metabolic deficits, leaving room for a specific amyloid or tau PET tracer. The aim of this study was to compare ASL-based to FDG-PET-based Z-score maps in patients with neurodegenerative dementia, using visual assessment.

## Materials and methods

Data from two cohorts were collected from separate study sites, one in Amsterdam, The Netherlands, and one in Uppsala, Sweden. Each cohort consisted of patients with a clinical diagnosis of either AD or FTD, and a control group. All subjects underwent MRI including a T1-weighted morphological sequence and a pseudocontinuous ASL perfusion sequence. On separate occasions within 6 months, all subjects underwent FDG-PET of the brain.

The Amsterdam patient cohort was enrolled from the VU University Medical Centre outpatient memory clinic. Prior to inclusion, the patients had received clinical probability diagnoses after standardized dementia work-up including medical history, extensive neuropsychological assessment, physical examination, blood tests, lumbar puncture and neuroimaging. All patients fulfilled criteria for clinical diagnosis of either AD [19] or behavioural variant FTD [20]. The Amsterdam control group was recruited from subjects with subjective memory complaints without verified cognitive or relevant psychiatric disorders. Controls underwent standardized dementia screening, had clinical assessments within normal range [14] and were selected to have normal CSF-aβ1-42 to exclude preclinical AD.

The Uppsala patient cohort was enrolled from the Uppsala University Hospital memory clinic. Prior to inclusion, the patients had received clinical probability diagnoses after standardized dementia work-up including medical history, neuropsychological assessment, physical examination, blood tests and neuroimaging. All patients fulfilled criteria for clinical diagnosis of AD [19], behavioural variant FTD [20] or semantic dementia [21]. The Uppsala control group was recruited through public advertising. The controls were interviewed and examined to rule out cognitive and relevant neurological and psychiatric pathology, and had clinical assessments within the normal range [22].

In total, 45 patients (25 AD and 20 FTD) and 38 controls were included (total *n* = 83). The study was approved by the local Medical Ethics Review Committees at the respective sites, and all subjects provided written informed consent.

### MRI protocol

All subjects were scanned using a 3T MRI scanner with an 8-channel head coil (Amsterdam: Signa HDxt; Uppsala: Achieva) with a protocol that included a 3D T1-weighted gradient echo sequence and pseudo-continuous ASL. In Amsterdam, the ASL pulse-sequence employed a 3D FSE readout with background suppression; post-label delay = 2.0 s; TR = 9 ms; TE = 4.8 s; spiral readout with 8 arms × 512 samples; 36 × 5.0 mm axial slices; 3.2 × 3.2 mm^2^ in-plane resolution; reconstructed pixel size = 1.7 × 1.7 mm^2^. In Uppsala the ASL pulse-sequence used a 2D GRE readout with background suppression: TR = 4,100 ms, TE = 14 ms, FOV = 230 × 230 mm, pixel size = 2.75 × 2.75 mm, 30 dynamic scans, 20 slices of 5 mm, flip angle 90°. The labeling duration was 1.65 s, and post-labeling delay 1.6 s. The multi-slice 2D imaging readout leads to an effective delay of 1.6 s for the first (inferior) slice, which increases to 2.3 s for the last acquired (superior) slice.

Acquisition time was approximately 4 min at both sites. No vascular gradient was used.

### FDG-PET protocol

On subsequent occasions (mean interval 73 days), the subjects underwent PET examinations after injection of ^18^F-FDG (FDG). The Amsterdam protocol included a 15-min scan starting 45 min after injection of 2.31 MBq/kg FDG (average dose 187 MBq), using a Gemini TF 64 PET-CT or ECAT Exact HR+ PET scanner (13 patients + three controls, and 17 patients + seven controls, respectively).

The Uppsala protocol included a 10-min scan starting 35 min after injection of 3 MBq/kg FDG (average dose 236 MBq), using a Discovery ST PET/CT scanner on all patients and 24 controls. Five of the controls were scanned on an ECAT Exact HR+ PET scanner.

Further details regarding the image acquisition have been previously described for both sites [14, 22, 23].

### Image post-processing

From the raw ASL data, perfusion maps were created (in Amsterdam: directly from the scanner; in Uppsala: using Nordic ICE version 3.0.0 Beta). The relative perfusion maps (relCBF) were spatially normalized in a two-step procedure using FSL (http://fsl.fmrib.ox.ac.uk/fsl). First, the individual images were linearly spatially normalized to the individual 3DT1 using FLIRT (part of FSL). Then, the non-linear transformation of the 3DT1 data to MNI space, which was obtained using the standard SPM processing pipeline, was applied, resulting in spatially normalized relCBF maps in MNI space. Next, mean and standard deviation relCBF parametric maps were calculated separately for the Amsterdam and Uppsala data. Finally, individual Z-score maps were calculated using the control data from each site, respectively.

The FDG-PET data from each subject was intensity normalized using a whole-brain reference value, calculated as the mean intensity of all voxels within the brain having an intensity of ≥ 0.8 of the maximum. Then, Z-score maps were produced using the same principle and computing pipeline as for the ASL images. Thus, two Z-score maps were created for each subject using the same post-processing procedure, one based on ASL-perfusion and one based on FDG-metabolism (166 maps from 83 subjects).

### Interface

All images were presented using nora, an in-house developed interface software (http://www.nora-imaging.com), providing all relevant features of a medical imaging viewer, including sagittal, transversal and coronal views and scrolling through the slices of all views. Thresholded Z-scores (Z-maps) were shown as overlays in a dichromatic colour scale, overlaid on an averaged and heavily smoothed grey scale brain image without specific anatomical information, to avoid assessment bias based on atrophy patterns. The Z-score thresholds used in the overlay were 2.0 (lower) and 6.0 (upper) by default and manually adjustable by the readers.

### Blinding

The Z-maps of patients and controls from both modalities were presented randomly and blinded with regard to diagnosis and modality used to construct the image. The readers neither had access to other images such as original PET or MRI images nor to any clinical information.

### Instruction tutorial

Prior to assessments, an instruction tutorial was available to the readers, including typical cases of AD and FTD. These additional images had been post-processed in the same way as the 83 subjects included in the study, and were presented in the same interface, with instructions and comments on how to conduct the assessments. An overview publication on deficit patterns was also added [4]. The purpose was to familiarize the readers with the graphical appearance of the current interface and to promote a common assessment strategy.

### Reading

Four experienced readers with specific interest in dementia imaging visually assessed and rated all images, two nuclear medicine physicians (NT, TD) and two neuroradiologists (KE, FB). The nuclear medicine trained readers had 10 and >20 years of experience of evaluation of dementia images, respectively, and the neuroradiologists had 11 and >20 years, respectively. Each reader assessed each Z-map and classified it as either a healthy control or patient, with a confidence level (range 0–4) representing how convincing the control/patient discrimination was. For images rated as pathological, the readers also made differential diagnostic decisions between AD and FTD.

### Statistical methods

Contingency tables were constructed with kappa values and Chi-squared tests. Proportion tests were used to compare the performance results listed in Tables 3 and 4. Inter-reader agreement per modality was evaluated pairwise for all readers using Cohen’s kappa and collectively for all readers using Fleiss’ kappa. Wilcoxon’s matched pairs test was used to compare the confidence levels. Graph production and statistical calculations were made using Dell Statistica, version 13, and IBM SPSS, version 23.

## Results

The Amsterdam patient cohort contained 18 patients with AD and 12 patients with FTD (*n* = 30, mean age 62.8 years), and the control group contained ten subjects (mean age 56.6 years). In one case, the spatial registration proved insufficient during post-processing, and this case was excluded from further analysis.

The Uppsala patient cohort contained seven patients with AD and eight with FTD (*n* = 15, mean age 66.7), and the control group contained 29 healthy individuals age-matched to the patients (mean age 67.8). In two healthy controls, the time between MRI and PET exceeded 6 months. In those cases the inclusion procedure, including interview, Mini-Mental State Examination (MMSE) and neurological examination, was subsequently repeated to ensure unchanged neurological and cognitive status.

Characteristics of the patients and controls are provided in Table 1.

**Table 1:** Subject characteristics by centre and diagnostic category. In total, 45 patients and 38 controls were included

	Amsterdam			Uppsala		
	AD *N* = 18	FTD *N* = 12	Controls *N* = 9	AD *N* = 7	FTD *N* = 8	Controls *N* = 29
Age, years (SD)	64 (8)	61 (8)	57 (9)	65 (9)	68 (10)	68 (7)
Gender (% male)	61%	42%	89%	57%	61%	52%
MMSE (SD)	24 (4)	24 (4)	27 (3)	24 (4)	28 (2)	29 (1)
Scan interval in months (SD)	2.1 (1)	2.2 (2)	1.4 (2)	1.9 (3)	2.6 (2)	2.9 (5)

*AD* Alzheimer’s disease, *FTD* frontotemporal dementia, *ASL* arterial spin labeling, *PET* positron emission tomography, *MMSE* Mini-Mental State Examination,

Mean values and standard deviations (SDs) are given

Scan interval is in months and represents the time between the ASL scan and the FDG-PET scan

**Fig. 1:**
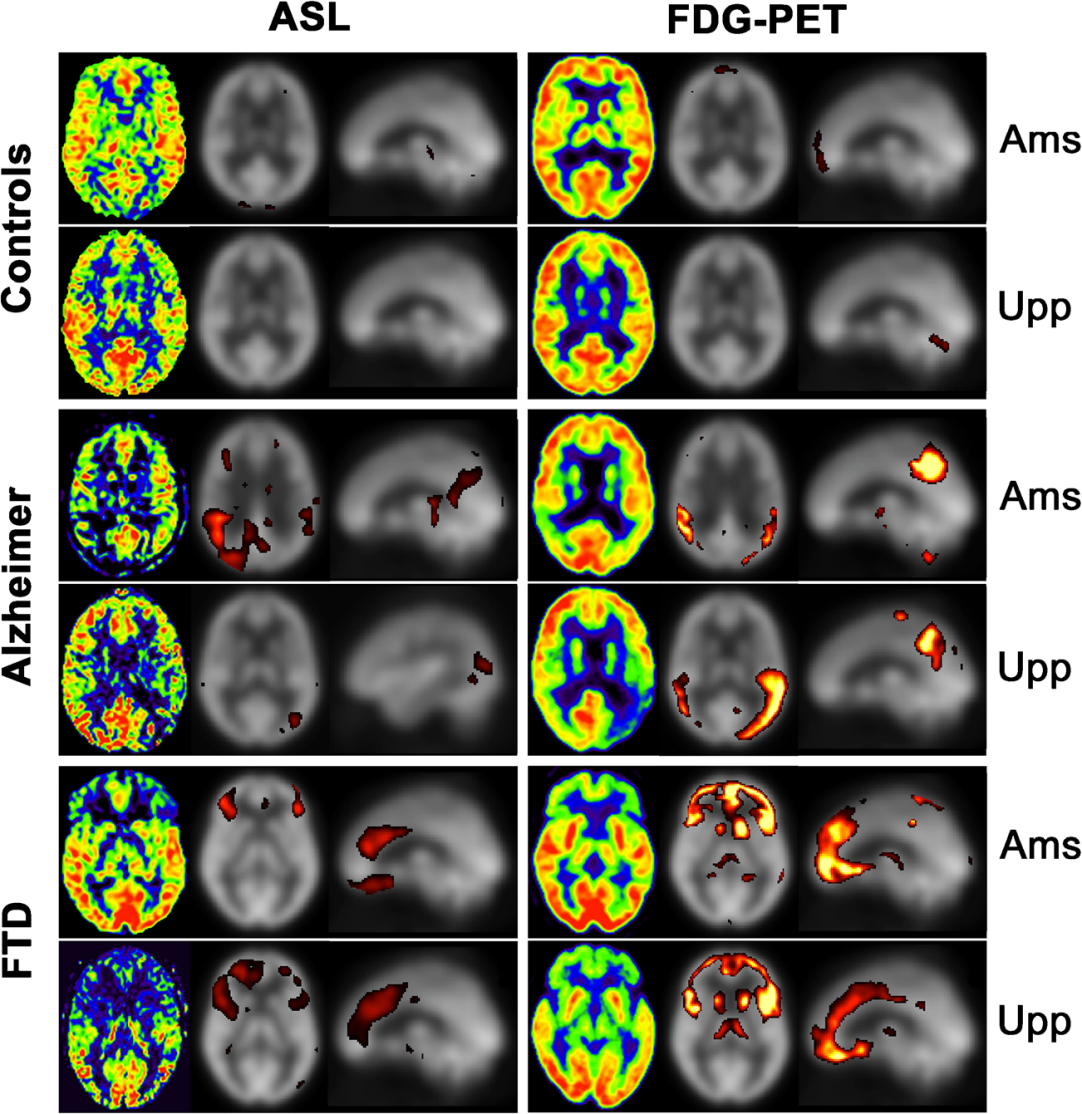
Representative subjects. Sample images from one control, one AD patient and one FTD patient from each site. Images are shown pairwise from the separate sites. ASL- and FDG-based images are shown in separate columns. The leftmost image in each box is an axial slice from the original image shown in a manually windowed Osirix rainbow scale. The middle and rightmost images in each box are axial and sagittal Z-maps created from the respective modality, with thresholded overlays shown in a dichromatic colour scale with the default lower and upper Z-score thresholds of 2.0 (red) and 6.0 (yellow). *AD* Alzheimer’s disease, *FTD* frontotemporal dementia, *ASL* arterial spin labeling, *Ams* Amsterdam, *Upp* Uppsala

Representative subjects are shown in Fig. 1, with the images from separate sites shown pairwise, and ASL-based and FDG-PET-based images placed columnwise for comparison.

### Patient versus control distinction

In a first step, we analysed the readers’ performance in distinguishing between patients and controls during assessments. Contingency tables of distinctions for all assessments are shown by modality in Table 2. Performance results and comparisons are listed in Table 3.

**Table 2:** Contingency table of patient/control distinction for all assessments (four raters × 83 maps), by modality. One data point was lost from the FDG-PET data during extraction (n = 331 instead of 332)

	ASL-based		FDG-PET-based	
Image rated as:	Controls	Patients	Controls	Patients
Normal	128	84	82	8
Pathological	24	96	69	172
	*n*: 332		*n*: 331	
	*K*: 0.364		*K*: 0.515	
	*p*: < 0.001		*p*: < 0.001	

K represents the Cohen kappa value of each contingency table

**Table 3:** Patient/control performance results by modality

Measure	ASL	FDG-PET	*p* value	
Sensitivity	53% (53%)	**96%** (99%)	<0.001	
Specificity	**84%** (85%)	54% (54%)	<0.001	
Negative predictive value	60% (61%)	**91%** (97%)	<0.001	
Positive predictive value	**80%** (81%)	71% (76%)	0.007	
Accuracy	68% (68%)	**77%** (80%)	0.008	
Uncertain cases, controls	7%	**25%**	<0.001	1)
Uncertain cases, patients	8%	7%	0.741	1)
Inter-reader agreement	0.58	0.38	N/A	2)
Unanimous distinction	66%	57%	0.235	3)
Unanimous correct distinction	49%	52%	0.607	4)
Distinction of AD cases	57%	**95%**	0.002	5)
Distinction of FTD cases	49%	**96%**	<0.001	5)
Higher confidence score	**125**	87	<0.001	6)

Values that were significantly higher than the corresponding value from the other modality have been marked in bold. The performance results in parentheses show the results after exclusion of uncertain cases

1 Rate of cases with confidence level <2

2 Fleiss’ kappa describing inter-reader agreement for all readers collectively

3 ‘Unanimous distinction’ means that all readers agreed on the patient/control distinction

4 All readers agreed on the *correct* distinction

5 The rate of patients from each diagnosis category that were correctly identified as patients

6 The number of instances with higher confidence score than the corresponding image from the other modality, examined with Wilcoxon matched pairs

*AD* Alzheimer’s disease, *FTD* frontotemporal dementia, *ASL* arterial spin labeling, *PET* positron emission tomography

For ASL-based maps, kappa values for pairwise inter-reader agreement were 0.71, 0.70, 0.67, 0.54, 0.54 and 0.40, and the total kappa for all readers was 0.58. For FDG-PET-based maps, kappa values for pairwise agreement were 0.51, 0.51, 0.39, 0.37, 0.36 and 0.33, and the total kappa for all readers was 0.38. No systematic differences were identified between the readers with a background in nuclear medicine compared to the neuroradiologists.

As shown in Table 3, ASL-based images yielded higher specificity, positive predictive value, inter-reader agreement and confidence scores. FDG-based images yielded higher sensitivity, negative predictive value and accuracy. However, the FDG-based images of controls contained a large number of uncertain cases. Exclusion of uncertain cases resulted in modest improvements in both modalities, shown in parentheses in the tables.

### Diagnosis-specific analysis

In a second step, we analysed the readers’ performance in assessing the correct differential diagnosis (AD vs. FTD). Results are provided in Table 4.

**Table 4:** Differential diagnosis performance results by modality

Measure	ASL	FDG-PET	*p* value	
Correct differential diagnosis	76% (80%)	84% (86%)	0.168	1)
Unanimous differential diagnosis	24%	**60%**	<0.001	2)
Correct diagnosis rate in AD cases	89%	82%	0.482	3)
Correct diagnosis rate in FTD cases	56%	**87%**	0.030	3)

Values that were significantly higher than the corresponding value from the other modality have been marked in bold. The performance results in parentheses show the results after exclusion of uncertain cases

1 Rate of correct differential diagnosis among true positive cases

2 All readers gave correct differential diagnosis

3 Rate of correct diagnosis among true positive cases

*AD* Alzheimer’s disease, *FTD* frontotemporal dementia, *ASL* arterial spin labeling, *PET* positron emission tomography

The patient groups were examined separately. ASL yielded a higher rate of correct differential diagnosis in AD cases compared to FTD cases (89% vs. 56%, *p* < 0.001). FDG-PET yielded similar results between patient groups (Table 3).

The cohorts were also examined separately. The rate of false-positive findings was higher in ASL images from the Uppsala cohort compared to the Amsterdam cohort (20% vs. 3%, *p* = 0.015). This difference was not evident in FDG-PET-based images (47% vs. 40%, *p* = 0.599). In other respects, the cohorts were similar, with no other difference reaching statistical significance.The results are provided in Table 5.

**Table 5:** Comparison between sites. There was a significantly higher false-positive rate in Uppsala controls compared to Amsterdam controls (*p* = 0.015). In other respects, results were similar between sites

Measure	Modality	Amsterdam	Uppsala	*p* value
False-positive rate	ASL	3%	20%	0.015
	FDG-PET	40%	47%	0.60
Distinction of AD cases	ASL	58%	54%	0.67
	FDG-PET	94%	96%	0.68
Distinction of FTD cases	ASL	46%	53%	0.52
	FDG-PET	96%	97%	0.81
Correct diagnosis rate in AD	ASL	86%	100%	0.12
	FDG-PET	81%	85%	0.62
Correct diagnosis rate in FTD	ASL	59%	53%	0.70
	FDG-PET	87%	87%	0.98

*AD* Alzheimer’s disease, *FTD* frontotemporal dementia, *ASL* arterial spin labelling, *PET* positron emission tomography

**Fig. 2:**
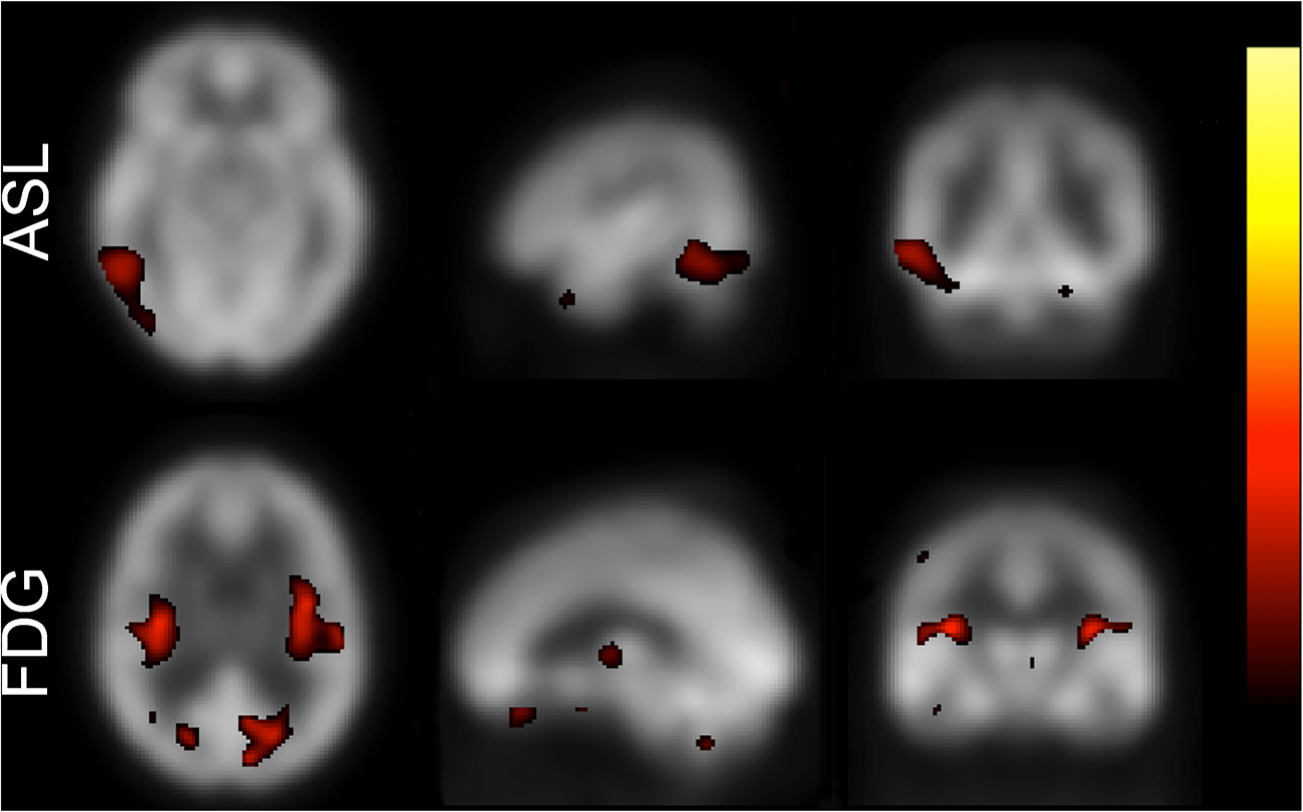
Healthy control with false-positive findings. Both the ASL-based and FDG-PET-based Z-score maps show false-positive Z-score patterns representative for the respective modality in this study. The clusters at the junction between grey and white matter in the lower row were present in several of the FDG-PET images of healthy controls, contributing to the number of uncertain cases, false-positive assessments and limited inter-rater agreement. The ASL-based map from this particular healthy control was read as AD by one reader, and the FDG-PET-based images as AD by two readers and FTD by one reader. The Z-maps are shown with thresholded overlays in a dichromatic colour scale with the default lower and upper Z-score thresholds of 2.0 (red) and 6.0 (yellow). *ASL* arterial spin labeling, *FTD* frontotemporal dementia, *PET* positron emission tomography, *AD* Alzheimer’s disease

A sample healthy control with false-positive findings is shown in Fig. 2. Both the ASL-based and FDG-PET-based Z-maps from this subject show false-positive patterns representative for the respective modality in this study.

**Fig. 3:**
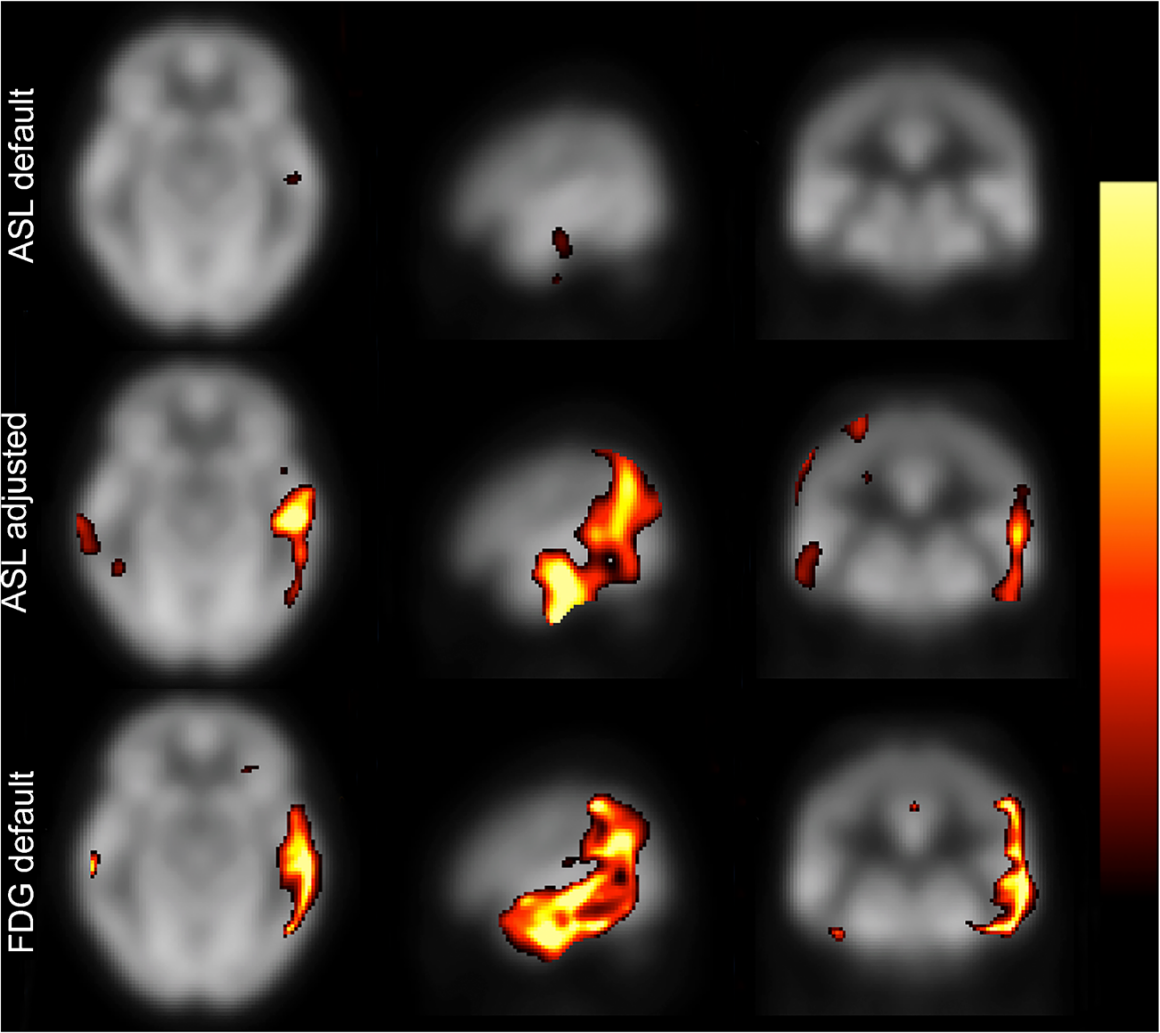
Representative discordant case. The ASL-based image of this AD case (Age: 67 years, MMSE: 25) was assessed as false negative by three of four readers, while the corresponding FDG-PET image was unanimously correctly diagnosed. The top row shows the ASL-based image with the default Z-score thresholds (lower 2.0, upper 6.0). The middle row shows the same image after threshold adjustment (0.77, 1.87). The bottom row shows the FDG-PET-based images of the same subject, with default threshold settings (2.0, 6.0). This is a representative case of a disease-typical deficit pattern evident on ASL-based images only after individually adjusting the threshold. Images are shown in a dichromatic colour scale ranging from red (lower threshold) to yellow (upper threshold). *ASL* arterial spin labeling, *AD* Alzheimer’s disease, *MMSE* Mini-Mental State Examination, *FTD* frontotemporal dementia, *PET* positron emission tomography

A representative discordant patient is shown in Fig. 3. The ASL-based map of this AD patient was assessed as false negative by three readers, while the FDG-PET-based map was correctly diagnosed by all readers. Several false-negative ASL cases in the study had such disease-typical deficit patterns that were less evident, or not at all evident, with the default settings, but could be revealed by individually adjusting the Z-score threshold.

## Discussion

The main finding in this study is that ASL-based Z-maps can be visually assessed with high specificity and positive predictive value in patients with AD and FTD. Sensitivity and accuracy were lower than in corresponding FDG-PET-based images. ASL can easily be added to the routinely performed MRI protocol of a dementia-workup, and creating Z-maps constitutes a relatively minor additional effort. The high specificity and positive predictive value of ASL indicates that an abnormal finding can provide a specific diagnosis with little additional effort. If ASL is negative or inconclusive but the clinical suspicion persists, the addition of FDG-PET could be considered due to its excellent sensitivity and superior accuracy.

The lower sensitivity of ASL in this study may imply that regional perfusion decrease is evident at a later stage than regional decrease of glucose metabolism. Since the ASL scans were acquired prior to FDG-PET, there is a theoretical possibility that disease progression between scans contributed to the difference, but this was considered unlikely. Sensitivity is also associated with the Z-score threshold, as discussed below.

ASL was more efficient in diagnosing AD cases compared to FTD, whereas FDG-PET showed no difference between patient groups. This could possibly represent a more disease-specific change of perfusion in AD than in FTD, as previously described [24]. This issue should be addressed in a larger study with a specific approach. Differential diagnosing was less robust in ASL than in FDG-PET.

When study sites were compared, a higher rate of false-positive findings was found among the ASL images in the Uppsala cohort. This could be caused by a higher mean age in the Uppsala control group, or by methodological differences between the ASL protocols at the two sites [11].

The rate of uncertain cases and false-positive results was higher in FDG-based images of healthy controls, associated with method-dependent false-positive Z-scores, as shown in Fig. 2. As a consequence, the FDG-based Z-maps showed poor specificity and inter-reader agreement. This is not in agreement with previous FDG-PET studies based on the 3D-SSP method [3, 25] and presumably depends on methodological differences – 3D-SSP maps are based on specific post-processing operations not used in this study. Instead, the current study used a voxel-wise comparison of all voxels after spatial normalization to directly compare ASL versus FDG-PET. Thus, the method used in this study is more vulnerable to registration mismatch and partial volume effects. 3D-SSP tools were not used because the available software packages are not constructed and optimized for ASL-MRI, which might bias the results. The authors would like to emphasize that the current method was intended for direct comparison between modalities only, not for clinical implementation. As a next step, assessing 3D-SSP analysis of ASL would be an interesting pursuit, with potential for clinical implementation [18].

Evaluation of ASL perfusion has several potential pitfalls such as artefacts and variability [26–28]. The quantification models currently available rely partly on approximations and assumptions, and development is on-going [29, 30]. There is considerable variance in cerebral perfusion levels in healthy controls, which makes cut-off values between normal and pathological perfusion difficult to establish [31–33]. The interindividual variance in ASL perfusion is large compared to the variance of SUV ratios in FDG-PET [31–33]. This is an important issue when comparing an individual subject to a control group of limited size.

While corrections for partial volume effects can be beneficial for optimizing the quantitative aspect of ASL, they are currently not used clinically in a standardized manner, and were not used in this study. In the clinical situation, the diagnostic physician can estimate the degree of partial volume effects by comparing the ASL scan to the structural images.

As shown in Fig. 3, the default threshold was not always suitable for the ASL-based images. In certain cases, adjusting the threshold revealed a distinct disease-typical pattern. The readers reported mostly using the default settings. Optimal thresholding for each subject was not systematically explored. This could be an interesting objective for a separate study with a different design. A larger normal database, preferably with subsampling of age and gender, would facilitate the distinction between normal and pathological. Plausibly, improved ASL sequences in the near future can provide more robust data with less variance.

Differences in scanning protocols between the sites could be a limitation due to possible bias, but was kept to a minimum by using the controls from each site as separate reference groups when constructing the Z-maps. The most important difference was the use of 2D versus 3D readout of the ASL-scans. Previous comparisons between 2D and 3D ASL protocols (similar to those used in this study) have shown regional differences in CBF, but similar total grey matter CBF, as well as similar coefficients of variance [34]. Multisite ASL studies have been considered problematic due to site dependence [11, 35]. The highly similar findings between sites in this study suggest that valid results are possible with different ASL protocols.

Another limitation is the sizes of the cohorts and control groups. The ideal control group would be larger and perfectly matched to the patients in gender and age, or large enough to sub-sample accordingly. Using subjects with subjective cognitive symptoms as controls, as done in the Amsterdam cohort, could be a potential confounding factor, but the rate of false positives was lower compared to the Uppsala cohort. The patient groups from the respective sites had slightly different compositions (Table 1), but the potential impact on the results was considered minor.

## Conclusions

ASL perfusion-based Z-score maps can be used as a diagnostic tool in patients with suspected neurodegenerative disease. In the current study, ASL provided high specificity and positive predictive value as a stand-alone sequence. In future studies, the performance can potentially be improved with implementation in a 3D-SSP software tool, and combined with morphological MRI data. FDG-PET might be reserved for those cases where ASL is negative and high clinical suspicion persists.

## Abbreviations

3D-SSP: Three-dimensional stereotactic surface projection
AD: Alzheimer’s disease
ASL: Arterial spin labeling
FTD: Frontotemporal dementia

## References

[1] Prince MWA, Guerchet M, Ali G, Wu Y, Prina M (2015) World Alzheimer report 2015. Alzheimer´s Disease International (ADI), London. Available via http://www.alz.co.uk/research/WorldAlzheimerReport2015.pdf. Accessed 11 Feb 2016

[2] Jack CR, Knopman DS, Jagust WJ (2013). Tracking pathophysiological processes in Alzheimer's disease: An updated hypothetical model of dynamic biomarkers. Lancet Neurol.

[3] Drzezga A (2009). Diagnosis of Alzheimer's disease with [18F]PET in mild and asymptomatic stages. Behav Neurol.

[4] Brown RK, Bohnen NI, Wong KK, Minoshima S, Frey KA (2014). Brain PET in suspected dementia: Patterns of altered FDG metabolism. Radiographics.

[5] Shivamurthy VK, Tahari AK, Marcus C, Subramaniam RM (2015). Brain FDG PET and the diagnosis of dementia. AJR Am J Roentgenol.

[6] Du AT, Jahng GH, Hayasaka S (2006). Hypoperfusion in frontotemporal dementia and Alzheimer disease by arterial spin labeling MRI. Neurology.

[7] Raji CA, Lee C, Lopez OL (2010). Initial experience in using continuous arterial spin-labeled MR imaging for early detection of Alzheimer disease. AJNR Am J Neuroradiol.

[8] Wolk DA, Detre JA (2012). Arterial spin labeling MRI: An emerging biomarker for Alzheimer's disease and other neurodegenerative conditions. Curr Opin Neurol.

[9] Binnewijzend MA, Kuijer JP, van der Flier WM (2014). Distinct perfusion patterns in Alzheimer's disease, frontotemporal dementia and dementia with Lewy bodies. Eur Radiol.

[10] Xekardaki A, Rodriguez C, Montandon ML (2015). Arterial spin labeling may contribute to the prediction of cognitive deterioration in healthy elderly individuals. Radiology.

[11] Alsop DC, Detre JA, Golay X et al (2015) Recommended implementation of arterial spin-labeled perfusion MRI for clinical applications: A consensus of the ISMRM perfusion study group and the European consortium for ASL in dementia. Magn Reson Med 73(1):102–116 10.1002/mrm.25197 PMC4190138 24715426

[12] Chen Y, Wolk DA, Reddin JS (2011). Voxel-level comparison of arterial spin-labeled perfusion MRI and FDG-PET in Alzheimer disease. Neurology.

[13] Musiek ES, Chen Y, Korczykowski M (2012). Direct comparison of fluorodeoxyglucose positron emission tomography and arterial spin labeling magnetic resonance imaging in Alzheimer's disease. Alzheimers Dement.

[14] Verfaillie SC, Adriaanse SM, Binnewijzend MA (2015). Cerebral perfusion and glucose metabolism in Alzheimer's disease and frontotemporal dementia: Two sides of the same coin?. Eur Radiol.

[15] Ishii K, Kono AK, Sasaki H (2006). Fully automatic diagnostic system for early- and late-onset mild Alzheimer's disease using FDG PET and 3D-SSP. Eur J Nucl Med Mol Imaging.

[16] Lehman VT, Carter RE, Claassen DO (2012). Visual assessment versus quantitative three-dimensional stereotactic surface projection fluorodeoxyglucose positron emission tomography for detection of mild cognitive impairment and Alzheimer disease. Clin Nucl Med.

[17] Minoshima S, Frey KA, Koeppe RA, Foster NL, Kuhl DE (1995). A diagnostic approach in Alzheimer's disease using three-dimensional stereotactic surface projections of fluorine-18-FDG PET. J Nucl Med.

[18] Takahashi H, Ishii K, Hosokawa C (2014). Clinical application of 3D arterial spin-labeled brain perfusion imaging for Alzheimer disease: Comparison with brain perfusion SPECT. AJNR Am J Neuroradiol.

[19] McKhann GM, Knopman DS, Chertkow H (2011). The diagnosis of dementia due to Alzheimer's disease: Recommendations from the National Institute on Aging-Alzheimer's Association workgroups on diagnostic guidelines for Alzheimer's disease. Alzheimers Dement.

[20] Rascovsky K, Hodges JR, Knopman D (2011). Sensitivity of revised diagnostic criteria for the behavioural variant of frontotemporal dementia. Brain.

[21] Neary D, Snowden JS, Gustafson L (1998). Frontotemporal lobar degeneration: A consensus on clinical diagnostic criteria. Neurology.

[22] Fallmar D, Lilja J, Velickaite V (2016). Visual Assessment of Brain Perfusion MRI Scans in Dementia: A Pilot Study. J Neuroimaging.

[23] Ossenkoppele R, Zwan MD, Tolboom N (2012). Amyloid burden and metabolic function in early-onset Alzheimer's disease: Parietal lobe involvement. Brain.

[24] Steketee RM, Bron EE, Meijboom R (2016). Early-stage differentiation between presenile Alzheimer's disease and frontotemporal dementia using arterial spin labeling MRI. Eur Radiol.

[25] Berti V, Pupi A, Mosconi L (2011). PET/CT in diagnosis of dementia. Ann N Y Acad Sci.

[26] Deibler AR, Pollock JM, Kraft RA, Tan H, Burdette JH, Maldjian JA (2008). Arterial spin-labeling in routine clinical practice, part 1: Technique and artifacts. AJNR Am J Neuroradiol.

[27] Deibler AR, Pollock JM, Kraft RA, Tan H, Burdette JH, Maldjian JA (2008). Arterial spin-labeling in routine clinical practice, part 2: Hypoperfusion patterns. AJNR Am J Neuroradiol.

[28] Grade M, Hernandez Tamames JA, Pizzini FB, Achten E, Golay X, Smits M (2015). A neuroradiologist's guide to arterial spin labeling MRI in clinical practice. Neuroradiology.

[29] Heijtel DF, Mutsaerts HJ, Bakker E (2014). Accuracy and precision of pseudo-continuous arterial spin labeling perfusion during baseline and hypercapnia: A head-to-head comparison with (1)(5)O H(2)O positron emission tomography. NeuroImage.

[30] Heijtel DF, Petersen ET, Mutsaerts HJ (2016). Quantitative agreement between [(15) O]H2 O PET and model free QUASAR MRI-derived cerebral blood flow and arterial blood volume. NMR Biomed.

[31] Chen JJ, Rosas HD, Salat DH (2011). Age-associated reductions in cerebral blood flow are independent from regional atrophy. NeuroImage.

[32] Preibisch C, Sorg C, Forschler A (2011). Age-related cerebral perfusion changes in the parietal and temporal lobes measured by pulsed arterial spin labeling. J Magn Reson Imaging.

[33] Henriksen OM, Larsson HB, Hansen AE, Gruner JM, Law I, Rostrup E (2012). Estimation of intersubject variability of cerebral blood flow measurements using MRI and positron emission tomography. J Magn Reson Imaging.

[34] Mutsaerts HJ, Steketee RM, Heijtel DF (2014). Inter-vendor reproducibility of pseudo-continuous arterial spin labeling at 3 Tesla. PLoS One.

[35] Mutsaerts HJ, van Osch MJ, Zelaya FO (2015). Multi-vendor reliability of arterial spin labeling perfusion MRI using a near-identical sequence: Implications for multi-center studies. NeuroImage.

